# NK Cells: A Double-Edged Sword in Chronic Hepatitis B Virus Infection

**DOI:** 10.3389/fimmu.2013.00057

**Published:** 2013-03-01

**Authors:** Mala K. Maini, Dimitra Peppa

**Affiliations:** ^1^Division of Infection and Immunity, University College LondonLondon, UK

**Keywords:** NK cells, hepatitis B virus, TRAIL, interferon-gamma, IL-10, CD56^bright^, IFN-alfa, liver damage

## Abstract

There is natural enrichment of NK cells in the human liver and this intrahepatic predominance underscores their potential importance in the control of infections with hepatotropic viruses such as hepatitis B virus (HBV). The contribution of innate components during chronic HBV infection has been a relatively under-investigated area. However, recent data have highlighted that NK cells are capable of exerting antiviral and immunoregulatory functions whilst also contributing to the pathogenesis of liver injury via death receptor pathways. We will present an overview of current knowledge regarding the complex biology of NK cells in the context of their antiviral versus pathogenic role in chronic hepatitis B as a clinically relevant avenue for further investigation.

## Introduction

The study of NK cells during infection with the hepatotropic hepatitis B virus (HBV) provides a useful opportunity to consider the function of these important immune effector cells within the unique liver environment. Despite the availability of a preventative vaccine, HBV continues to cause persistent infection in an estimated 400 million people worldwide. The virus itself is non-cytopathic, but triggers immune responses resulting in persistent inflammation and progressive fibrosis in the infected liver. These immune-mediated pathological processes ultimately lead to cirrhosis and hepatocellular carcinoma, resulting in more than a million HBV-related deaths annually. Although the introduction of new antiviral agents with a high genetic barrier to resistance has improved the treatment of HBV, sustained cure rates are still low (Papatheodoridis et al., 2008). The inherent capacity of the immune system to efficiently contain HBV in acutely infected adults who resolve infection provides a strong rationale for the development of new treatments based on immunotherapeutic boosting. However, this requires a careful dissection of the multifaceted immune response, to identify which components can be harnessed to control viremia and/or its pathological consequences without exacerbating disease. Here we will consider the current evidence in favor of protective or pathogenic roles for NK cells in HBV infection.

## NK Cells in Context: The Liver Microenvironment

In order to understand the role of NK cells in HBV infection it is necessary to first consider some specialized features of the liver microenvironment, well-known for its tolerogenic properties. This state of “immune privilege” prevents an overwhelming response to the innocuous antigens that the liver is constantly exposed to from the gastrointestinal tract, but provides an advantageous setting for those pathogens able to infect the liver to establish chronicity. Hepatic tolerance is maintained by a number of aspects of the local immunological environment including the cytokine and nutrient milieu, and influences of the target hepatocytes and other specialized resident cell types (Crispe, 2009; Protzer et al., 2012).

**Figure 1:**
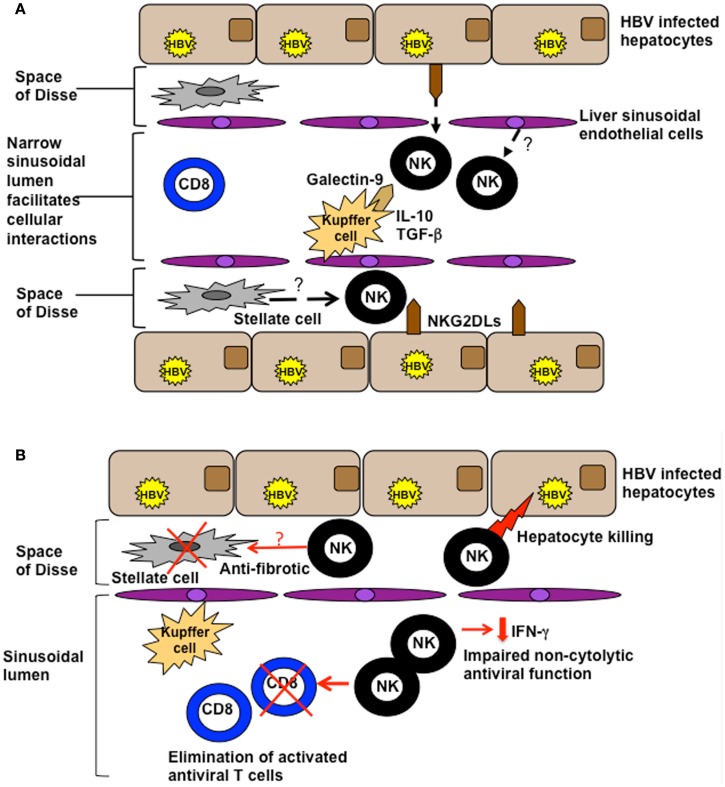
**(A)** Potential influences of the hepatic milieu on NK cells. The liver sinusoidal endothelial network is extensive and narrow, with sluggish blood flow encouraging intimate contact between circulating and resident cell types. NK cells are exposed to a milieu rich in IL-10 and TGF-β and can interact with ligands on a number of specialized resident liver cells such as galectin-9 on Kupffer cells and NKG2D on hepatocytes. They may also be influenced by inhibitory/stimulatory ligands expressed by liver sinusoidal endothelial cells or stellate cells. **(B)** Functions of NK cells in the HBV-infected liver. NK cells can utilize death ligands to kill hepatocytes (contributing to liver damage) and HBV-specific T cells (curtailing antiviral immunity). They exhibit impaired non-cytolytic antiviral function (reduced IFN-γ production), but may serve a protective function to limit liver fibrosis by killing activated stellate (pro-fibrotic) cells.

NK cell biological function is tightly regulated by the balance of signals provided by their diverse array of cell surface receptors, combined with the cytokine milieu. In the liver, their activation is likely to by heavily influenced by the ligands they encounter on the cells lining the extensive sinusoidal network (Figure 1A). The potential for liver sinusoidal endothelial cells and Kupffer cells (sinusoidal-resident macrophages) to display relevant NK cell ligands is an area just beginning to be investigated. For example Tim-3, upregulated on NK cells in chronic HBV infection (CHB), can down-modulate their function (Ju et al., 2010); its ligand, galectin-9, is strongly expressed by Kupffer cells (Nebbia et al., 2012). Kupffer cells also have the potential to influence NK cells through their cytokine production; they are one of many intrahepatic cell types able to produce immunosuppressive cytokines such as IL-10 and TGF-β that can tolerize local NK cells (Tu et al., 2008), as discussed further below. NK cells can in addition make direct contact with infected hepatocytes, either through fenestrations in the sinusoidal lining or after migrating into the parenchyma. Thus intrahepatic NK cell function may be further shaped by the balance of signals received from hepatocytes, with their very low levels of MHC Class I but potential to upregulate cellular stress ligands (Chen et al., 2007).

One of the most striking differences between the immune composition of the liver and the blood is the proportion of NK cells. In the healthy liver, the percentage of NK is typically increased more than threefold compared to the periphery, accounting for around a third of the intrahepatic mononuclear cell population (Doherty et al., 1999). Liver NK cells also show phenotypic and functional characteristics that are distinct from their circulating counterparts. In particular, intrahepatic NK cells are more activated and the majority have a CD56^bright^ phenotype, thought to be an earlier stage of differentiation than the CD56^dim^ phenotype predominating in the periphery (Burt et al., 2009; Shi et al., 2011). Even the CD56^dim^CD16+ fraction of intrahepatic NK cells express lower levels of KIRs than this subset in the periphery, which may limit their capacity to be adequately licensed (Burt et al., 2009). The fetal liver is a site of NK cell generation, raising the possibility that some NK cells arise in the adult liver, in line with their immature phenotype. The derivation of adult intrahepatic NK cells has been addressed by an interesting study documenting the rapid re-population of liver grafts with recipient NK cells and their precursors (Moroso et al., 2011). These data suggest that NK cell precursors derived from the bone marrow are recruited from the circulation and take on the “signature” of hepatic NK cells once resident in the liver. This hepatic NK cell enrichment is maintained in the inflammatory infiltrate characteristic of HBV infection (Sprengers et al., 2006; Dunn et al., 2007). The chemotactic signals regulating the influx and retention of human hepatic NK cells and NK cell precursors remain to be defined. Particularly pertinent to this is the recent finding in murine models that the chemokine receptor CXCR6 retains a population of NK cells in the liver that mediate long-lived “memory” responses to viruses and haptens (Paust et al., 2010).

## The Antiviral Role of NK Cells in HBV Infection

There is an accumulating body of evidence supporting an antiviral role for NK cells, reinforced by the elaborate mechanisms employed by a number of viruses for NK cell immune evasion (Martin et al., 2002; Orange et al., 2002; Khakoo et al., 2004; Lee et al., 2007). More recent studies have identified associations between control of human persistent viral infections such as HIV and HCV and particular genes for KIRs (a highly polymorphic family of NK receptors) and the HLA molecules they interact with (Martin et al., 2002; Khakoo et al., 2004). Associations between KIRs and viral control may be the result of differential efficiency of NK effector function (Alter et al., 2007); the idea that NK cells can exert immune pressure on persistent viruses such as HIV is supported by evidence of KIR-associated viral adaptation at a population level (Alter et al., 2011).

NK cells have the capacity to exert antiviral activity in HBV infection through direct effects or indirectly, by modulating T cell responses. Whilst NK cells can promote T cell responses through their production of cytokines like IFN-γ, emerging data are highlighting their capacity to conversely limit antiviral responses by deletion or inhibition of T cells (Waggoner et al., 2010, 2012; Lang et al., 2012). Direct NK cell effects could involve lysis of infected hepatocytes through granzyme/perforin or death receptor pathways. However viral clearance through these mechanisms would only be achieved at the expense of death of infected hepatocytes (see section on liver damage below). Non-cytolytic mechanisms of HBV clearance through cytokines like IFN-γ (Guidotti et al., 1996) are therefore thought to play a critical role in mediating viral control in the infected liver, whilst preserving the integrity of this vital organ. The contribution of NK cells to these mechanisms in the acutely and chronically HBV-infected liver remains controversial.

The antiviral potential of NK cells has been demonstrated in animal models of HBV infection, with NK cells efficiently inhibiting HBV replication in transgenic mice (Kakimi et al., 2000) and making a contribution to clearance in the hydrodynamic injection model of acute HBV (Yang et al., 2002). In chimpanzees, NK cells were initially implicated by the finding that the first phase of non-cytolytic clearance of HBV-infected hepatocytes was accompanied by an increase in intrahepatic IFN-gamma and TNF-alpha (Guidotti et al., 1999). However, subsequent experiments showed a critical role for T cells rather than NK cells in HBV control in this model (Thimme et al., 2003). Studies of patients in the pre-clinical ramp-up phase of acute HBV infection revealed an increase in the number of circulating NK cells (Webster et al., 2000; Fisicaro et al., 2009) but their activation and effector function was suppressed as viral load increased and only peaked once viremia had resolved (Dunn et al., 2009). This inhibition of NK cell activation and effector potential showed an inverse temporal correlation with induction of the immunosuppressive cytokine IL-10, raising the possibility that HBV can actively evade immune responses (Dunn et al., 2009). No data are available from humans immediately after HBV inoculation regarding the involvement of NK cells before detectable viral replication. However woodchucks infected with high doses of the closely related woodchuck hepatitis virus showed upregulation of the NK cell activating receptor NKp46 immediately after infection (Guy et al., 2008), suggesting that NK cells may make their major contribution to HBV control in the earliest lag phase of infection.

In CHB, persistent, high-level viremia, and the tolerizing liver environment combine to drive profound exhaustion of antiviral T cells. In the presence of a disabled T cell response, ineffective viral control, and ongoing liver inflammation, it is plausible that the large number of activated NK cells could perform a compensatory antiviral function. However our recent data reveal that NK cells in this setting instead play a key role in disarming adaptive immunity. We observed that activated HBV-specific T cells, particularly those in the intrahepatic compartment, upregulate death receptors that render them susceptible to elimination by NK cells (Peppa et al., 2013; Figure 1B). Furthermore, we and other groups have shown that NK cells in patients with CHB become defective in their production of IFN-γ (Oliviero et al., 2009; Peppa et al., 2010; Tjwa et al., 2011), making them ineffective at exerting direct non-cytolytic antiviral functions and at promoting T cell responses. We postulated that this selective defect in NK cell function may be attributable to the influence of IL-10 and TGF-β in the liver, since it was restored following *in vitro* blockade of these immunosuppressive cytokines (Peppa et al., 2010). IL-10 is induced in flares of CHB (Das et al., 2012), as in acute infection, and can recapitulate a selective defect in NK cell IFN-γ production (Peppa et al., 2010). Evidence from murine models suggests that these immunosuppressive cytokines may impair NK cell function by modifying their receptor expression. IL-10 was found to contribute to the regulation of liver NK cells by maintaining a higher percentage of the hyporesponsive NKG2A^+^Ly49^−^ subset of NK cells (Lassen et al., 2010). In patients with CHB, the partial recovery of NK cell IFN-γ production upon antiviral therapy has been linked to the downregulation of NKG2A (Tjwa et al., 2011). Impaired functionality of NK cells in CHB has also been attributed to the capacity of TGF-β to downregulate the NKG2D/DAP10 and 2B4/SAP pathways (Sun et al., 2012). In a further recent study, HBV modulation of pDC–NK cell crosstalk was found to contribute to impaired IFN-γ production by NK cells (Shi et al., 2012), highlighting the importance of reciprocal interactions with other innate cells.

## Pathogenic Roles of NK Cells in the HBV-Infected Liver

Hepatitis B virus is a non-cytopathic virus which mediates liver disease by the immune responses it triggers. Elegant experiments in the HBV transgenic mouse model demonstrated a role for the non-specific infiltrate recruited in the liver at the time of peak inflammation in acute hepatitis (Ando et al., 1993). Analogous findings came from the study of livers from patients with persistent HBV infection, in whom an infiltrate of non-antigen specific lymphocytes was found to be associated with liver inflammation (Maini et al., 2000). One of the largest constituents of the inflammatory infiltrate in HBV transgenic mice was NK1.1+CD3− NK cells, with a 10- to 12-fold increase in their numbers compared to baseline (Kakimi et al., 2001). An infiltration of NK cells has been shown to be associated with Pseudomonas induced hepatotoxicity (Beraza et al., 2009), MCMV induced hepatitis in mice (Salazar-Mather et al., 1998), and persistent HCV infection (Nuti et al., 1998).

The large number of activated intrahepatic NK cells could contribute to HBV-induced liver damage through a number of effector mechanisms. As discussed above, circulating NK cells preferentially maintain their cytolytic compared to non-cytolytic capacity in HBV infection (Oliviero et al., 2009; Peppa et al., 2010). Intrahepatic NK cells have been less thoroughly studied in this regard but a recent study has suggested that they are likewise biased to cytotoxicity in patients with active HBV-related inflammation compared to those with no biochemical evidence of liver damage, or to healthy controls (Zhang et al., 2011). However some studies suggest hepatocytes may be relatively resistant to perforin/granzyme-mediated cytotoxicity (Tay and Welsh, 1997; Kafrouni et al., 2001) and mouse models have supported a role for death ligands from the TNF superfamily in mediating liver damage through the induction of hepatocyte apoptosis (Galle et al., 1995; Balkow et al., 2001; Zheng et al., 2004). Hepatocyte apoptosis is increasingly recognized to play a part in the initiation of hepatic inflammation (Malhi and Gores, 2008); this is likely to be particularly relevant in the context of CHB since infected apoptotic cells have been shown to instruct an immunogenic rather than tolerogenic immune response (Stockinger, 2009).

One death ligand implicated in liver damage is TRAIL (Zheng et al., 2004), which murine liver NK cells were originally noted to express in the setting of tumor metastases (Takeda et al., 2001). Human NK cells were reported to express minimal amounts of TRAIL in both the circulation and liver of healthy individuals (Ishiyama et al., 2006), but work from our lab revealed that NK cells could upregulate this ligand in the setting of HBV-related liver inflammation (Dunn et al., 2007). Unlike NK cell capacity for the production of cytokines and cytotoxic mediators, which vary according to the *in vitro* stimulus employed to elicit their production, the expression of death ligands such as TRAIL could be assessed directly *ex vivo*. TRAIL was found to be most highly expressed on the CD56^bright^ subset of NK cells that is preferentially expanded and activated in the liver. A death receptor for TRAIL, TRAIL-R2 (DR5), which had been previously thought to be restricted to tumor tissue, was found to be upregulated on the surface of hepatocytes in liver biopsies from patients with CHB. NK cells from patients with HBV-related liver inflammation but not from healthy controls, were able to induce apoptosis of primary human hepatocytes. Blocking studies suggested this was partially attributable to TRAIL but pointed to the involvement of additional pathways (Dunn et al., 2007).

The Fas pathway has previously been implicated in hepatocyte apoptosis and HBV-related liver disease (Ogasawara et al., 1993; Galle et al., 1995). A more recent study provided evidence that DC-activated NK cells are capable of inducing HBV-infected hepatocyte degeneration in a humanized mouse model through the Fas/FasL system (Okazaki et al., 2012). Supporting a role for Fas mediated liver injury, the expression of FasL on NK cells in HBV patients correlated with progression of acute-on-chronic liver failure (Zou et al., 2010). However, a direct role for NK cells mediating liver damage via the Fas pathway has not been fully established in human infection with HBV. NK cells are also a potent source of TNF, a pro-inflammatory cytokine that has been shown to augment liver injury (Mizuhara et al., 1994). Hepatic TNF and TNFR1 expression are enhanced in CHB (Mizuhara et al., 1994; Fang et al., 1996), but again the capacity of these interactions to kill hepatocytes, and the involvement of NK cells, remains to be demonstrated in CHB.

An interesting area requiring further dissection is how the complex array of activatory and inhibitory NK cell receptors, the cytokine milieu, and viral factors combine to regulate these potential pathways of liver damage and viral elimination. Major activatory NK cell receptors like NKG2D and NKp46 are likely to play a key role in directing NK cell function in CHB. In support of this, a recent study knocking down multiple NKG2D ligands on hepatocytes was able to protect against fulminant hepatitis (Huang et al., 2013). In the case of the TRAIL pathway, we found that IFN-α was able to potently upregulate NK cell ligand expression (Dunn et al., 2007), in line with subsequent studies suggesting that this is a major mechanism of action of therapeutic IFN-α in the setting of HBV (Micco et al., 2013) and HCV (Stegmann et al., 2010; Ahlenstiel et al., 2011). The enhancement of NK cell effector function during IFN-α treatment was associated with an induction of NKp46 expression (Micco et al., 2013). The cytokines IL-2 (Ishiyama et al., 2006) and IFN-γ (Tu et al., 2008) have also been shown to be able to induce TRAIL, with a recent study demonstrating a role for monocyte-derived IFN-γ in driving NK cell TRAIL expression (Sarhan et al., 2012). The sensitivity of hepatocytes to TRAIL-mediated apoptosis is controlled by NEMO, the regulatory subunit of the IKK complex mediating NF-kappaB activation (Beraza et al., 2009) and can be enhanced by their upregulation of death-inducing receptors, that may be promoted by IL-8 (Dunn et al., 2007), TGF-β (Saitou et al., 2005), and HBV proteins (Janssen et al., 2003; Liang et al., 2007). Likewise, susceptibility of hepatocytes to TNF-induced death has recently been shown to be modulated by viral infection, which sensitizes them to caspase-mediated apoptosis induction (Wohlleber et al., 2012). TNF can also cooperate with FasL to induce hepatocyte apoptosis by activating Bim and Bid (Schmich et al., 2011). Taken together, these studies suggest that NK cell-mediated liver damage requires a combination of factors enhancing NK cell activation with others able to sensitize hepatocytes.

## Conclusion: Promoting Protective Roles of NK Cells in the HBV-Infected Liver

Activated NK cells are abundant in the HBV-infected liver and may play a role in early viral containment. With the progression of chronic infection, NK cells, like T cells, are subjected to the tolerizing effects of hepatic ligands and cytokines, skewing them toward functional defects that limit their antiviral efficacy (Peppa et al., 2010; Figure 1B). Conversely, NK cells are well-placed to mediate pathogenic functions in CHB, promoting liver damage (Dunn et al., 2007), and further constraining antiviral immunity by deleting HBV-specific T cells (Peppa et al., 2013). However, blocking key mediators of such effects would not be without risk since it appears that NK cells may utilize these same pathways to exert important protective effects in the liver.

NK cells have been shown to limit fibrosis in rodent models by killing stellate cells (the major hepatic profibrogenic population) in a TRAIL and NKG2D-dependent manner (Radaeva et al., 2006). A recent study supported this role for NK cells in HCV and suggested that the anti-fibrotic effects of IFN-α therapy may be partially attributable to its potential to boost NK cell-mediated killing of stellate cells (Glassner et al., 2012). Hepatic NK cells may also be able to produce IL-22, which has the capacity to be hepatoprotective, limiting tissue damage (Zenewicz et al., 2007). It will be important to investigate whether subsets of NK cells in CHB serve anti-fibrotic and/or hepatoprotective functions. Recent data also point to a critical role for NK cells in tumor protection in the setting of CHB (Kamimura et al., 2012), where the development of hepatocellular carcinoma is a major threat.

Further studies are required to better understand the factors triggering and mediating the opposing roles of NK cells in CHB to allow these to be successfully exploited for therapeutic targeting. Dissecting pathways of human hepatic NK cell development and education will increase our understanding of the mechanisms involved in shaping NK cell responsiveness in the liver. Future attempts to manipulate NK cell immunity in the setting of hepatotropic viral infections would need to be carefully timed and tailored to block pathogenic effects whilst harnessing vital protective functions.

## Conflict of Interest Statement

Mala K. Maini filed a patent for TRAIL blocking in viral hepatitis, received an unrestricted educational grant from BMS and sat on advisory boards for Roche, Transgene, ITS. Dimitra Peppa declares that the research was conducted in the absence of any commercial or financial relationships that could be construed as a potential conflict of interest.
